# Breastfeeding in Neonates Admitted to an NICU: 18-Month Follow-Up

**DOI:** 10.3390/nu14183841

**Published:** 2022-09-16

**Authors:** Rozeta Sokou, Stavroula Parastatidou, Georgios Ioakeimidis, Evangelia-Filothei Tavoulari, Athanasia Makrogianni, Elina Isaakidou, Nicoletta Iacovidou, Aikaterini Konstantinidi

**Affiliations:** 1 Neonatal Intensive Care Unit, “Agios Panteleimon” General Hospital of Nikea, 3 D. Mantouvalou Str., Nikea, 18454 Piraeus, Greece; 2 Neonatal Department, Aretaieio Hospital, School of Medicine, National and Kapodistrian University of Athens, 11528 Athens, Greece

**Keywords:** breastfeeding, neonates, preterm neonates, breastfeeding support, lactation, hospitalized neonates

## Abstract

Introduction: The admission of neonates to Neonatal Intensive Care Units (NICUs) has been identified as a primary inhibiting factor in the establishment of breastfeeding. The aims of this study were to (1) estimate the prevalence and duration of breastfeeding in infants/toddlers who had been admitted to an NICU in Greece and (2) to investigate factors, associated with the NICU stay, which affected the establishment and maintenance of breastfeeding in infants/toddlers previously admitted to the NICU. Materials and methods: Data for this cohort study were retrieved from interviews with mothers of infants/toddlers who had been admitted to our NICU as neonates during the period of 2017–2019. Interviews were conducted based on a questionnaire regarding the child’s nutrition from birth to the day of the interview, including previous maternal experience with breastfeeding. Information related to the prenatal period, gestation age, delivery mode, duration of NICU stay, and neonatal feeding strategies during their hospital stay were recorded. Results: The response rate to the telephone interviews was 57%, resulting in 279 mother–infant pairs being included in this study. The results showed that 78.1% of children received maternal milk during their first days of life. Of all infants, 58.1% were exclusively breastfed during their first month, with a gradual decrease to 36.9% and 19.4% by the end of the third and sixth months of life, respectively. The prevalence of breastfed children reached 14.7% and 7.5% at the ages of twelve and eighteen months, respectively. In the multivariate analysis, prematurity emerged as an independent prognostic factor for the duration of exclusive and any breastfeeding (aHR 1.64, 95% CI: 1.03–2.62; and 1.69, 95% CI: 1.05–2.72, respectively; *p* < 0.05). Additionally, the nationality of the mother, NICU breastfeeding experience, the administration of maternal milk during neonatal hospital stay, and previous breastfeeding experience of the mother were independent prognostic factors for the duration of breastfeeding. Conclusions: Although breastfeeding is a top priority in our NICU, the exclusive-breastfeeding rates at 6 months were quite low for the hospitalized neonates, not reaching World Health Organization (WHO) recommendations. Mothers/families of hospitalized neonates should receive integrated psychological and practical breastfeeding support and guidance.

## 1. Introduction

For optimal health outcomes, the World Health Organization recommends exclusive breastfeeding for the first 6 months of life, followed by the appropriate introduction of complementary foods with continued breastfeeding to two years and beyond [1,2,3].

Multiple recent publications report the short-term and long-term advantages of maternal milk for preterm neonates. Maternal milk contains the optimal immunologic, anti-oxidative, and growth factors for various neonatal systems [4].

Feeding with human milk (HM) from the neonate’s own mother reduces the risk for short-term and long-term morbidity and, subsequently, the cost of care of ill preterm and full-term neonates [5]. Regarding preterm neonates, higher HM doses are correlated with a lower risk of enteral feeding intolerance, late sepsis, chronic pulmonary disease, retinopathy of prematurity, neurocognitive impairment, and less hospital re-admissions by the ages of 18–30 months [6,7,8,9,10,11,12,13,14,15,16]. 

Almost 10–12% of neonates born in the United States are preterm, and admission to NICUs is necessary for many of them [17]. Approximately 10% of full-term neonates require more-advanced-than-usual medical care, and a large proportion of them is also admitted to NICUs. Hospitalized neonates represent a population with a higher risk of adverse short-term and long-term outcomes than healthy full-term neonates [18,19,20]. Admission to NICU has been suggested as a primary inhibiting factor in establishing breastfeeding, with neonates admitted to NICUs presenting lower rates of breastfeeding than healthy neonates [21,22,23,24,25]. Probable causes of this phenomenon include the separation of the mother and the neonate; the stress and anxiety of the mother, which may result in depressive disorder; and the clinical status of the mother and/or the neonate [26,27,28,29]. Until now, in Greece, the direct impact of postnatal mother–neonate separation in establishing and maintaining breastfeeding in neonates admitted to NICUs is largely unknown and needs to be further investigated. 

The aims of this study were to (1) assess the prevalence and duration of breastfeeding in infants/toddlers who had been admitted to a Greek NICU and (2) to assess the probable effect of certain factors associated with the NICU stay on the rate, establishment, and duration of breastfeeding in infants/toddlers previously admitted to the NICU. 

## 2. Materials and Methods 

Data for this retrospective study were collected from interviews with mothers of infants/toddlers who were admitted to our NICU as neonates during 2017–2019. This research study followed the STROBE checklist (Supplementary Table S1). 

### 2.1. Definitions

Exclusive Breastfeeding (EBF): The Infant Only Receives Maternal Milk and No Other Liquids, with the Exception of Vitamins, Rehydration Solutions, Minerals, and Medicines

The vast majority of neonates hospitalized in NICUs receive parenteral nutrition during the first days of life. In this study, the breastfeeding status was assessed following the achievement of full enteral feeding. Maternal milk during NICU stay was fortified in very-low-birthweight neonates; however, these neonates were included in the exclusively breastfed group if they had not received any formula milk. 

Any Breastfeeding (BF): Breastfeeding, Either Exclusive or Partial Breastfeeding, Supplemented with Formula Milk or Other Foods.

### 2.2. Breastfeeding Support and Promotion in NICU

The NICU of General Hospital “Agios Panteleimon” is a perinatal center of the 2nd Health District, which includes West Attica areas and the Aegean Sea islands. A high percentage of the neonates admitted to our NICU are transferred from remote areas, with a significant impact on breastfeeding availability and establishment in this population. Maternal milk is valuable for the care/treatment provided in an NICU; therefore, the establishment of breastfeeding in hospitalized neonates is a primary target of our NICU. With all neonatal admissions, as soon as possible following birth, parents are informed by appropriately educated personnel on the advantages of breastfeeding, on ways to maintain lactation, and on the storage and transfer conditions of maternal milk. Relevant information materials with detailed instructions are handed out to the parents upon the admission of their neonate. When the maternal–neonatal state allows it, skin-to-skin contact is recommended and encouraged (twice daily for at least thirty minutes). All mothers are educated and supported to breastfeed throughout the day, provided this is permitted by the neonatal state, safely and successfully, under the supervision of experienced personnel. 

### 2.3. Inclusion Criteria

All neonates born during the time period from January 2017 until December 2019 who were admitted to our NICU were included in this study. 

### 2.4. Exclusion Criteria 

Neonates of families residing in refugee camps were excluded from the study due to difficulties in communication (in the Greek or English language) with the mother/father and problems with the completion of the questionnaire. 

Neonates with congenital anomalies directly affecting enteral feeding, neonates for whom breastfeeding was absolutely contraindicated, and all the neonates who died in the NICU were excluded from the study. 

### 2.5. Measurement

A structured questionnaire was created in order to retrieve data with regards to the nutrition of the child from birth to the interview, as well as maternal breastfeeding experience previous to this child. The percentage of breastfed infants, the percentage of exclusively breastfed infants, and the percentage of infants who were still breastfed at three, six, nine, twelve, and >eighteen months of age were documented. Furthermore, data regarding demographic characteristics of the mothers, previous breastfeeding experience, the timing of solid foods introduction, and breastfeeding experience during the NICU stay of the neonate were recorded (questionnaire data are presented in detail in the Supplementary Materials). Information on the prenatal period, gestation length, delivery mode, the duration of hospital stay, and the feeding of the neonates during their hospital stay was retrieved from medical records. 

### 2.6. Questionnaire Design

The questionnaire was designed to allow us to estimate the basic breastfeeding frequency indexes suggested by the WHO [2]. 

The questionnaire was pilot-tested in 21 mothers to determine the time needed for completion, the degree of participant comprehension, and the sequence of questions. Subsequently, the questionnaire was revised based on pilot testing.

### 2.7. Interview

Contact details were retrieved through the medical files from the admission of neonates to the NICU. The study primarily included data from interviews with mothers. In case the mother did not speak Greek, the father could answer the interview questions in the presence of the mother. A telephone interview was conducted when the study infants were older than twelve months (March 2021–May 2021). The interview duration was approximately six to ten minutes. 

**Figure 1:**
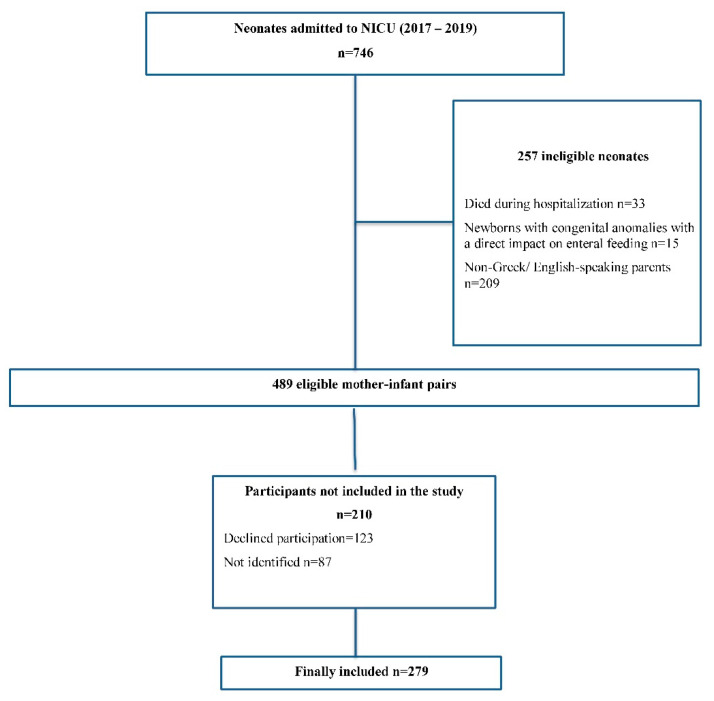
Flow chart of the study population.

The study was conducted according to the guidelines of the Declaration of Helsinki and was approved by the Institutional Review Board of Nikeaia General Hospital “Agios Panteleimon” (3/11, 22 January 2020). The interview was conducted following the verbal consent of the parent. Recruitment data are presented in a flow diagram (Figure 1).

### 2.8. Statistical Analysis

Before comparing the independent groups, data distribution was examined for the determination of the most appropriate analysis. Initially, data were visually assessed by comparing their histograms with the normal probability curve; then, the Kolmogorov–Smirnov test for normality was performed. Both assessments demonstrated that the data were not normally distributed. For the descriptive statistics of quantitative variables, median values and interquartile range were used. Absolute (Ν) and relative (%) frequencies were used to describe qualitative variables. The non-parametric Mann–Whitney U test was applied for the comparison of the quantitative variables (which were non-normally distributed) between two groups. For the comparison among more than two groups, the non-parametric Kruskal–Wallis criterion was used. The duration of breastfeeding was assessed using the Kaplan–Meier survival estimator, with the cessation of exclusive and any breastfeeding being considered as the final events for the analysis. Infants who were breastfed at the end of the study period were labeled as censored. The duration of breastfeeding was defined as the number of months until the cessation of breastfeeding or from birth until the final date of follow-up. A Cox proportional hazards regression analysis was applied for the investigation of the simultaneous effect of several risk factors on the duration of breastfeeding. Covariate effects were considered using hazard ratios (HRs) and their 95% confidence intervals. A significance level of 0.05 was set (two-tailed significance levels). The SPSS 22.0 statistical program (IBM SPSS Statistics for Windows, Version 22.0. Armonk, NY, USA: IBM Corp.) was used for the statistical analyses. 

## 3. Results

### 3.1. Descriptive Results

The response rate to the telephone interviews was 57%, resulting in 279 mother–infant pairs being included in this study. A total of 255 mothers responded, 24 of whom had given birth to twins. Of all participating neonates, 56.3% were full-term, and 66.3% had been delivered via cesarean section; in total, 31.5% were inborn, while 68.5% were outborn. The median birthweight of our sample of infants was 2.700 g (1.960–3.250 g), and the median gestational age was 37 (34–38) weeks. Of the participating neonates, 25 (9%) were very-low-birthweight neonates; a total of 43 (15.4%) had a gestational age < 32 weeks, and 79 neonates (28.3%) were late preterm. A total of 21 neonates (7.5%) presented intrauterine growth restriction (IUGR), and 137 neonates (49.1%) had respiratory distress syndrome; in total, 67 neonates (24%) suffered from perinatal hypoxia, and 12 (4.3%) neonates were admitted for surgical purposes. Forty-two (15%) of the admitted neonates presented early-onset sepsis. Most of the participating mothers were Greek (72.4%), followed by Albanians (17.9%). The median length of stay in the NICU was 12 (7–23) days. The median time (months) to the initiation of infant formula or solids was 1 (0–5) and 6 (5–6) respectively. Permanent residents of Attica accounted for 60.2% of our sample. Nearly half of the participants (42.7%) had previous breastfeeding experience, and only 4.7% had attended breastfeeding classes. Of the participating mothers, 144 (51.6%) were primigravidae, while 135 were multigravidae. Among the multigravid mothers, 119 (88.1%) had breastfeeding experience with a previous child. Detailed demographic characteristics’ data are presented in Table 1. 

### 3.2. Prevalence of Breastfeeding

#### 3.2.1. Exclusive Breastfeeding

The prevalence of exclusively breastfed infants was 58.1% for the first month of life and reduced to 36.9% by the end of the third month. By the end of the sixth month, only 19.4% of the infants were exclusively breastfed, with a gradual drop during the next months to reach 2.2% by the end of the eight month. 

#### 3.2.2. Any Breastfeeding 

The breastfeeding rate during the first month of life was quite high, reaching 78.1% and remaining at 47.7% until the completed third month of life. During the next months, a gradual decrease in breastfeeding prevalence was observed, reaching 32.6% by the end of the sixth month. The percentages of breastfed infants at the ages of nine, twelve, and eighteen months were 17.9%, 14.7%, and 7.5%, respectively. 

A shorter duration of exclusive breastfeeding was observed for preterm neonates compared with full-term neonates, and this difference was statistically significant (*p*-value < 0.05). The clinical characteristics of the study neonates are presented in Table 2. 

The data from the Kaplan–Meier survival analysis of breastfed preterm and full-term neonates are presented in Table 3.

**Figure 2:**
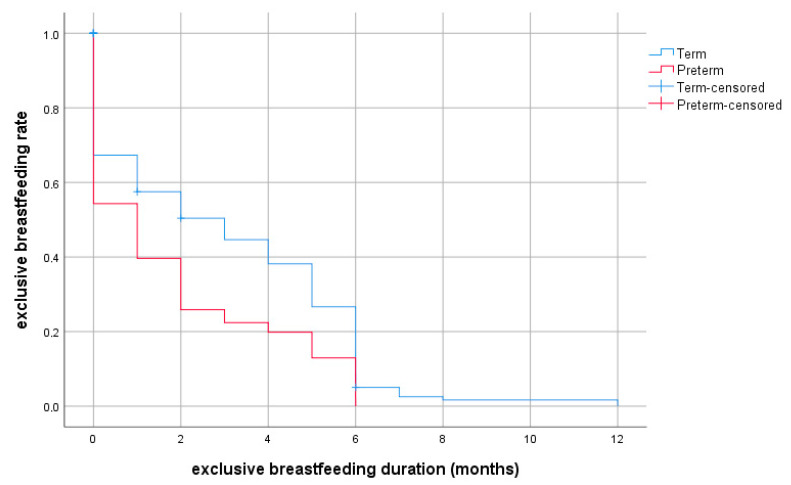
Probability of exclusive breastfeeding depending on duration (months, discrete survival curves) according to prematurity.

**Figure 3:**
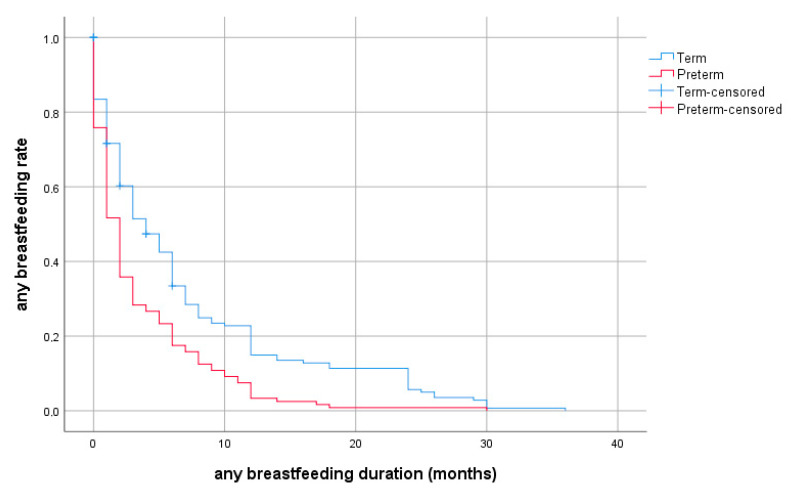
Probability of any breastfeeding depending on duration (months, discrete survival curves) according to prematurity.

The rates of exclusive breastfeeding and any breastfeeding at selected ages are provided in detail in Figure 2 and Figure 3 for preterm and full-term groups.

The statistical analyses (Table 1) indicated that neonates of Greek-origin mothers had significantly shorter breastfeeding duration than neonates of mothers of other nationalities. Neonates who had received maternal milk during their NICU stay or had been breastfed in the NICU and those whose mothers had previous breastfeeding experience or had attended breastfeeding classes had been breastfed for significantly longer than the remaining neonates. The delivery mode (cesarean section) and transportation of the neonate affected the duration of breastfeeding, as a shorter duration of exclusive and any breastfeeding was observed in these neonates, but not at a statistically significant degree (*p* > 0.05). A multivariable analysis was conducted to investigate the impact of various factors on the duration of exclusive and any breastfeeding. The duration (in months) of exclusive breastfeeding and the duration of any breastfeeding were the dependent variables, while gestational age, delivery mode, nationality (categorized as Greek and other), inborn/outborn, permanent residence, prematurity, hospital stay, previous breastfeeding experience, feeding with maternal milk during hospital stay, breastfeeding experience in the NICU, and maternal attendance of breastfeeding classes were the independent variables. The multivariable analysis indicated prematurity as an independent prognostic factor for the duration of exclusive and any breastfeeding, with preterm neonates presenting a higher hazard ratio of earlier breastfeeding cessation than full-term neonates (aHR 1.64, 95% CI:1.026–2.62; and 1.69, 95% CI:1.054–2.72, respectively; *p* < 0.05). Furthermore, NICU breastfeeding experience, maternal-milk administration during hospital stay, and previous breastfeeding experience were positively and strongly correlated with breastfeeding duration, as a lower hazard ratio of breastfeeding discontinuation was noted (*p* < 0.05). The attendance of breastfeeding classes by the mother was an independent prognostic factor strongly associated with the duration of breastfeeding (aHR 0.41, 95% CI: 0.218–0.77; *p* = 0.006) but did not seem to affect the duration of exclusive breastfeeding (aHR 0.76, 95% CI: 0.424–1.375; *p* = 0.37). The multivariable analysis did not reveal any statistically significant effect on the duration of exclusive or any breastfeeding for the remaining factors under investigation (Table 4 and Table 5).

## 4. Discussion

Breastfeeding practices in Greece have not been thoroughly investigated. This study assesses breastfeeding status in a Greek NICU, contributing to the recognition of factors that affect the prevalence, establishment, and maintenance of breastfeeding in neonates admitted to the NICU. The percentage of exclusive breastfeeding at the first and sixth months of age in our study was lower than that recommended by the WHO [2] and CDC [30]. This was consistent with findings of previous studies [22,31,32]. The exclusive-breastfeeding rates in our study infants were similar to the respective rates of the general Greek population [31,32]. Maternal nationality, breastfeeding during NICU stay, maternal-milk administration during NICU stay, and maternal experience of breastfeeding older children were significantly positively correlated with the duration of breastfeeding. Prematurity, on the other hand, was inversely correlated with breastfeeding duration, which is consistent with data from other countries [5,33]. 

In our cohort, the prevalence of exclusively breastfed infants during the first month of life was 58.1%. Dritsakou et al. [34] recruited 161 healthy pregnant women who attended prenatal breastfeeding classes and assessed the effect of maternal diet, personal traits, and the intention to breastfeed on the breastfeeding duration of neonates admitted to a Greek NICU. The authors reported that 81% of the study neonates were exclusively breastfed at discharge.

There is published evidence that preterm neonates tend to breastfeed less and for a shorter period of time than full-term neonates [35,36], and our findings are also in accordance with this. Consistently with previous studies [33,37], prematurity emerged as an adverse factor for exclusive and any breastfeeding in our study neonates. Preterm neonates are generally transferred to the NICU immediately after birth and are separated from their mothers, which results in the late initiation of breastfeeding [38]. The lower the gestational age is, the longer the neonate’s hospital stay is. Most preterm neonates below 34 weeks need to be fed via nasogastric tube due to sucking–swallowing incoordination. Neonates with respiratory distress, face anomalies, and central neural system disorders also require tube feeding, and the pumping of breast milk is necessary in these cases. The advantages of feeding preterm neonates with maternal milk, and especially colostrum, are paramount. However, and despite the significant efforts by the mothers and healthcare providers in NICUs, only around 30% of mothers giving birth to extremely low birthweight neonates manage to exclusively support the newborn with their milk during the first days of life [39,40]. The inability to ensure the required amount of milk to exclusively support the neonate is a primary aggravating factor for the psychology of the mother and may lead to the cessation of breastfeeding [41]. Medical personnel should focus on preterm and ill neonates, educating mothers to monitor daily lactation by completing a lactation diary, and intervene if needed for the optimization and promotion of breastfeeding in these neonates. The transfer of maternal milk by mothers and their families in order to feed the neonates during their NICU stay was associated with higher prevalence and duration of exclusive breastfeeding. Formula-feeding preterm neonates in the NICU was shown to affect the duration of exclusive breastfeeding following discharge [33]. In a national study in Denmark, it was observed that when mothers were allowed to visit and feed their preterm neonates in the NICU with a feeding cup or spoon, the hospital stay of these neonates was decreased. Moreover, mothers of neonates who had breastfed during their hospital stay continued breastfeeding for longer after discharge [42,43]. According to our findings, maternal-milk administration during hospital stay and breastfeeding experience in the NICU were prognostic independent variables for the duration of exclusive and any breastfeeding. Studies in multicultural societies demonstrated that refugees of any national group tended to maintain breastfeeding for longer than native mothers, even after the adjustment for socio-economic and demographic factors [44,45]. In our study population, the mother’s nationality was found to be an independent confounding factor for the duration of breastfeeding, with Greek mothers ceasing breastfeeding earlier than mothers of any other nationality. Previous breastfeeding experience was positively correlated with the duration of breastfeeding. In a large study in the Netherlands [46], a similar correlation was noted, although shortly after birth, firstborn children were more likely to be breastfed. This finding may be attributed to the fact that the reasons that led to the breastfeeding of the older child still existed for the younger newborn. In addition, the mother is more confident, has already practiced breastfeeding, and may be more knowledgeable regarding its advantages. It is well established that breastfeeding classes/seminars bear multiple benefits for both the mother and the neonate, as they offer, before labor, important information on the process and advantages of breastfeeding, leading to its successful initiation and establishment [47,48]. Despite the low rate in our sample, the attendance of breastfeeding classes seemed to have a positive effect on the duration of breastfeeding. 

Multiple studies have investigated risk factors for breastfeeding practices. A Lancet series in 2016 reported a wide range of historical, socioeconomical, cultural, and personal prognostic factors for breastfeeding practices [49]. There is evidence that cesarean section has a negative impact on the initiation and duration of breastfeeding, especially if it has been performed under general or spinal anesthesia [50,51]. This finding was attributed to the lower maternal prolactin levels, the post-operative pain and, particularly, to the delayed contact of the mother with the neonate. The labor mode was not associated with the duration of breastfeeding in our study. That could have been due to the fact that both preterm and full-term neonates who had been admitted to the NICU had failed to achieve the early skin-to-skin contact with their mothers and that the initiation of breastfeeding was delayed compared with healthy neonates. 

Recent studies focused on the association of inhibiting neighborhood factors on the duration of breastfeeding. It was reported that the dependence on public transportation and long distance commuting to the NICU negatively affected the frequency of maternal visits and the pumping and transport of maternal milk [52,53]. A large proportion of our NICU hospitalized neonates are transferred from distant areas of the country; however, neonatal transfer and permanent residence did not seem to impact the establishment of breastfeeding in our study. This could be explained by the practices of supporting and promoting neonatal feeding with maternal milk that are applied in our NICU. 

Maternal milk is the ideal nutrition for neonates and infants, protecting against infections and facilitating long-term health. Furthermore, it is a crucial element of public health, especially for preterm neonates (gestational age < 37 weeks) [54]. Breastfeeding bears immunological, nutritional, and neurodevelopmental benefits for preterm neonates. It is protective against necrotizing enterocolitis, bronchopulmonary dysplasia, and late sepsis [55,56,57,58]. Maternal-milk effects are dose-dependent. The quantity of maternal milk consumed by a neonate is inversely correlated with risk of death and necrotizing enterocolitis during the first 2 weeks of life [10]. Studies showed that high HM doses during the first 14–28 days of life are associated with a lower risk of various adverse outcomes in the NICU [7,10,11,12]. A research line indicated that it is the presence of bovine products (and not just the absence of feeding with HM) that negatively impacts intestinal permeability and colonization, rendering the association between HM and neonatal morbidity more complicated [6,16,59,60]. However, accumulating evidence suggests that bioactive HM components provide specific protection against morbidity through various mechanisms during different hospitalization periods in the NICU. Moreover, breastfeeding plays an important role on cognitive development, leading to a productive adulthood. Maternal milk includes long-chain polyunsaturated fatty acids, which promote brain growth. Research demonstrated that early visual acuity and cognitive functions are better developed in breastfed children [59,61,62]. Breastfeeding also appears to have a positive impact on infants’ emotional well-being. It helps establish mother–infant bonding, due to skin-to-skin contact, which allows the infant to smell, touch, and feel their mother. Breastfeeding is pivotal for both the mother and the infant, as their developing bond is critical for the individual and reflects on the whole family. It has been shown that breastfed infants have closer and more intimate relationships with other family members. All in all, breastfeeding contributes to the smooth emotional and social development of the infant [28,60]. NICU admission of the neonate results in physical and psychologic separation from the mother, a key factor responsible for the failure of breastfeeding [63]. Rooming-in is extremely fortifying for breastfeeding because it contributes to the development of a communication code, providing peace, protection, and safety to the neonate. Breastfeeding plays an important role in the prognosis of preterm and ill neonates. In addition to the optimal nutrition that covers their substantial needs for growth and development, breastfeeding is also therapeutic for preterm neonates [64]. Data on the benefits of HM use in NICUs are intriguing, yet the incorporation of this evidence in practices, policies, procedures, and parental educational materials is limited. HM feeding is still under-prioritized over other therapeutic interventions implemented in the NICUs. Scarce information and lactation induction practices for the optimization of breastfeeding are available to healthcare professionals in NICUs and to the families of the neonates [5]. 

This study had a few limitations. The study design did not contain extensive data on the feeding status of the infant and/or the timing of breastfeeding initiation during their NICU stay. In addition, other important factors possibly affecting the duration of breastfeeding following the discharge of the neonate, including maternal age, educational level, and socio-economic status, were not recorded. Feedback on the duration of breastfeeding was provided by parents, making information bias possible; for example, mothers could have responded based on social expectations rather than their actual experience. The questionnaire was not validated in an extended Greek breastfeeding population. Sample size was only 279 mother–infant pairs. The breastfeeding practices of participants in this study may not be representative of regional or national practices. Probable systematic bias deriving from these limitations should be taken into account. Further large-scale, well-designed studies are necessary before the generalization of these study results. 

## 5. Conclusions

Although breastfeeding support is a top priority in our NICU, the breastfeeding rates at six months for previously hospitalized infants were quite low compared with the standards set by the WHO. NICUs should promote breastfeeding, providing mothers/families with psychological support and comprehensive guidance on breastfeeding practices. The NICU environment is appropriate for educational interventions, as mothers are in close contact with healthcare professionals and have frequent access to lactation consultants and other breastfeeding resources. To better leverage this opportunity for the promotion of breastfeeding, healthcare professionals should identify mothers at a high risk of early breastfeeding cessation. Subsequently, educational and supportive interventions would need to be adjusted to overcome the obstacles impeding this subpopulation from breastfeeding. Evidence-based quality indicators are required for the comparative assessment of HM administration. The establishment of procedures that protect breastfeeding and the incorporation of lactation technologies that facilitate milk transportation are essential. An NICU is more than a treatment center for neonates; it is a living environment for newborns and their parents, with a focus on family-centered care.

## Figures and Tables

**Table 1:** Characteristics of the study population and median duration of any and exclusive breastfeeding (in months).

				Duration of Any Breastfeeding (Months)		Duration of Exclusive Breastfeeding (Months)	
				Median (IQR)	*p*-Value	Median (IQR)	*p*-Value
**GA (weeks)**		37	34–38	2 (1–6)		1 (0–5)	
**BW (g)**		2.700	1.960–3.250				
**Nationality, N and %**	Greek	202	72.4%	2 (0–60	0.001	1 (0–4)	0.008
	Albanian	50	17.9%	4.5 (1–12)		2.5 (0–6)	
	Other Balkan nationality *	8	2.9%	4 (1.25–21)		2 (0–5)	
	Indian	5	1.8%	9 (6–19)		5 (1.5–6)	
	Arab	7	5%	12 (0–120		6 (0–12)	
**Permanent Residence, N and %**	Attica	168	60.2%	2 (1–6.75)	0.998	1 (0–50	0.422
	Mainland Greece	70	25.1%	2 (0.75–6)		1.5 (0–5)	
	Islands	41	14.7%	2 (1–7.5)		2 (0–5)	
**Delivery mode,** **N and %**	Vaginal	94	33.7%	3 (0.75–8.25)	0.421	1.5 (0–5)	0.125
	Cesarean section	185	66.3%	2 (1–6)		1 (0–4)	
**Outborn,** **N and %**	Yes	88	31.5%	2 (0–6)	0.24	1 (0–4.75)	0.214
	No	191	68.5%	2 (0–6)		1 (0–5)	
**Maternal milk in NICU, N and %**	No	78	28%	0 (0–2)	0.000	0 (0–0.25)	0.000
	Yes	201	72%	4 (1–8.0)		2 (0–6)	
**NICU breastfeeding, N and %**	No	135	48.6%	1 (0–3)	0.000	0 (0–2)	0.000
	Yes	143	51.4%	6 (2.0–12)		3 (1–6)	
**Breastfeeding classes, N and %**	No	266	95.3%	2 (1–6)	0.19	1 (0.5)	0.327
	Yes	13	4.7%	3 (1–16.5)		2 (1–5)	
**Older child, N and %**	No	145	51.6%	2 (0.5–6)	0.116	1 (0–5)	0.197
	Yes	136	48.4%	3 (1–8)		1 (0–5)	
**Previous breastfeeding experience, N and %**	No	160	57.3%	2 (0–6)	0.000	1 (0–4)	0.041
	Yes	119	42.7%	4 (1–8)		2 (0–5)	

Abbreviations: GA, gestational age; BW, birthweight; IQR, interquartile range; * Bulgarian and Romanian nationalities.

**Table 2:** Clinical characteristics of preterm and full-term study neonates.

Term Neonates (N = 157)					Preterm Neonates (N = 122)				
	Mean	Median	Percentile 25	Percentile 95	Mean	Median	Percentile 25	Percentile 95	*p*-Value
GA	38	38	38	40	33	33	32	36	0.000
BW	3294	3190	2820	4000	1948	1950	1620	2850	0.000
Hospital stay (days)	11	8	6	32	32	21	14	115	0.000
Duration of any breastfeeding (months)	6	3	1	25	3	2	0	12	0.000
Duration of exclusive breastfeeding (months)	3	2	0	6	2	1	0	6	0.002

Abbreviations: GA, gestational age; BW, birthweight.

**Table 3:** Data on breastfeeding preterm and full-term neonates.

**Means and Medians for Survival Time—Breastfeeding Duration (Months)**									
**Neonates**	**Mean**				**Median**				***p*-Value**
	Estimate	Std. Error	95% Confidence Interval		Estimate	Std. Error	95% Confidence Interval		
			Lower Bound	Upper Bound			Lower Bound	Upper Bound	
**Full-term neonates**	6.875	0.703	5.497	8.253	3	0.597	1.831	4.169	0.00
**Preterm neonates**	3.416	0.44	2.553	4.279	2	0.22	1.569	2.431	
**Overall**	5.35	0.45	4.468	6.233	2	0.283	1.446	2.554	
**Means and Medians for Survival Time—Exclusive-Breastfeeding Duration (Months)**									
**Full-term neonates**	2.99	0.232	2.534	3.445	3	0.641	1.743	4.257	0.002
**Preterm neonates**	1.75	0.203	1.352	2.148	1	.	.	.	
**Overall**	2.441	0.162	2.124	2.758	1	0.277	0.457	1.543	

**Table 4:** Prognostic factors associated with duration of exclusive breastfeeding (Cox proportional hazards regression analysis, N = 279).

Variable in the Equation	Duration of Exclusive Breastfeeding					
	B	SE	*p*-Value	aHR	95.0% CI	
					Lower	Upper
**Preterm**	0.494	0.239	0.04	1.64	1.026	2.62
**Hospital stay (days)**	0.003	0.003	0.32	1.00	0.997	1.01
**Previous breastfeeding experience**	−0.192	0.08	0.02	0.83	0.705	0.97
**Maternal milk in NICU**	−0.485	0.191	0.01	0.62	0.424	0.90
**Breastfeeding in NICU**	−0.429	0.171	0.01	0.65	0.466	0.91
**Breastfeeding classes**	−0.27	0.3	0.37	0.76	0.424	1.375
**Gravida (<I)**	0.253	0.225	0.26	1.29	0.829	2.001
**GA (weeks)**	0.064	0.047	0.18	1.07	0.972	1.169
**BW (g)**	0.00	0.00	1.00	1.00	1	1
**Nationality (non-Greek)**	−0.343	0.161	0.03	0.71	0.518	0.973
**Permanent residence (Attica)**	−0.069	0.09	0.44	0.93	0.782	1.112
**Delivery mode (caesarian section)**	0.098	0.141	0.49	1.10	0.837	1.453

Abbreviations: B, beta coefficient; CI, confidence interval; SE, standard error; aHR, adjusted hazard ratio; GA, gestational age; BW, birthweight.

**Table 5:** Prognostic factors associated with duration of any breastfeeding (Cox proportional hazards regression analysis, N = 279).

Variable	Breastfeeding Duration					
	B	SE	*p*-Value	aHR	95.0% CI	
					Lower	Upper
**Preterm**	0.527	0.242	0.03	1.694	1.054	2.722
**Hospital stay (days)**	0.003	0.003	0.425	1.003	0.996	1.009
**Previous breastfeeding experience**	−0.228	0.088	0.009	0.796	0.67	0.945
**Maternal milk in NICU**	−0.753	0.198	0.000	0.471	0.319	0.694
**Breastfeeding in NICU**	−0.58	0.173	0.001	0.56	0.399	0.785
**Breastfeeding classes**	−0.893	0.322	0.006	0.41	0.218	0.77
**Gravida (<I)**	0.088	0.244	0.718	1.092	0.677	1.763
**GA (weeks)**	0.042	0.048	0.385	1.042	0.949	1.145
**BW (gr)**	0.00	0.00	0.495	1	1	1
**Nationality (non-Greek)**	−0.457	0.16	0.004	0.633	0.463	0.866
**Permanent residence (Attica)**	−0.028	0.092	0.763	0.973	0.813	1.164
**Delivery mode (cesarean section)**	0.28	0.145	0.053	1.324	0.997	1.757

Abbreviations: B, beta coefficient; CI, confidence interval; SE, standard error; aHR, adjusted hazard ratio; GA, gestational age; BW, birthweight.

## Data Availability

The data presented in this study are available on request from the corresponding author.

## Supplementary Materials

The following supporting information can be downloaded at: https://www.mdpi.com/article/10.3390/nu14183841/s1, Table S1: STROBE Statement—checklist of study.
